# Gas vs. Chloroform

**Published:** 1871-03

**Authors:** L. N. Hutchinson


### GAS *vs.* CHLOROFORM.

Eds. Dental Register:—I have recently seen it stated that "among the various anæsthetics in use, experience has proved that chloroform is the pleasantest, most convenient of administration, and as safe as any." My experience and observation corroborates the statement, and yet scarcely a week passess but some one tells us he has taken gas, or he has a friend who has taken it, and they could not be induced to take it again, because it made them sick, or produced very disagreeable sensations while administered. Many of our profession advertise the use of gas, and commend it as perfectly safe and harmless, when they must know, if they read the dental journals, that the reckless manufacture and use of it are more disastrous to health and life than chloroform or ether. The trouble is, they have cried out such vengeance against chloroform and ether, and have so lumbered their rooms with apparatus for the monster gas, that they must keep it before the people, or fail for an anæsthetic. Some have learned to be "just out of gas," or to have "broke" their "retort," and "will have to send for another," but they can give ether or chloroform! Oh, consistency thou art a jewel. But these combined with more than half our professors who do not use gas, together with all our medical men who do not use it either, we can safely say that in not more than one case in five is gas given, and my humble opinion is that the bad effects and fatalities are in favor of chloroform and ether. This I should attribute to impure *nitrous oxide* arising from incompetent manufacture, or miserable method of administering it. I have no choice of anæsthetic agents, provided they are pure, for the result to the human system is the same. Now if experience has proved that gas is no safer than any other agent, why lumber our parlors with apparatus for making it, and spend time and patience, and endanger our heads by bursting retorts, and in the end produce no better or pleasanter agent?

Be honest, then, and tell the community the truth in regard to all agents producing anæsthesia, and they will thank you for it, and the profession will be all the better for it too.

L. N. HUTCHINSON.

